# Associations of childhood-to-adulthood body size trajectories with dementia risk and brain structural imaging markers: a study from UK Biobank

**DOI:** 10.3389/fnut.2026.1764658

**Published:** 2026-07-28

**Authors:** Zihang Zhang, Pan Zhang, Yanyan Li, Jun Chu, Chengwei Su, Zhixin Hu, Houren Xiong, Gongxia Xu

**Affiliations:** 1 Department of Cardiovascular Surgery ICU, The First Affiliated Hospital of Anhui Medical University, Hefei, Anhui, China; 2 Department of Neurology, The First Affiliated Hospital of USTC, Division of Life Sciences and Medicine, University of Science and Technology of China, Hefei, Anhui, China; 3 Department of Cardiovascular Medicine, The First Affiliated Hospital of Anhui University of Traditional Chinese Medicine, Hefei, Anhui, China; 4 Respiratory Intensive Care Unit of Yijishan Hospital, Wannan Medical College, Wuhu, Anhui, China; 5 Department of Intensive Care Unit, Ding Yuan General Hospital, Dingyuan, Anhui, China; 6 Department of Liver Transplantation, The First Affiliated Hospital of USTC, Division of Life Sciences and Medicine, University of Science and Technology of China, Hefei, Anhui, China

**Keywords:** body mass index, brain structure, dementia, obesity, risk factors

## Abstract

**Background:**

Childhood and adulthood adiposity may influence dementia risk and brain structure; however, their life-course interplay and potential sex-specific effects remain incompletely understood.

**Methods:**

We analyzed 488,397 UK Biobank participants with self-reported comparative body size at age 10 (categorized as thinner, average, or plumper relative to peers) and measured adulthood BMI, followed prospectively for incident dementia. Cox models estimated hazard ratios (HRs) with 95% confidence intervals (CIs). A subsample (*n* = 45,759) underwent brain MRI.

**Results:**

Compared with average childhood body size, a plumper body size at age 10 was associated with a higher risk of all-cause dementia (HR 1.12, 95% CI 1.04–1.20), particularly among men (HR 1.15, 95% CI 1.04–1.28), whereas a thinner body size was not significantly associated with dementia risk. In adulthood, overweight was associated with lower risks of all-cause dementia (HR 0.86, 95% CI 0.81–0.92) and Alzheimer’s disease (HR 0.83, 95% CI 0.76–0.91), while obesity was associated with a higher risk of vascular dementia (HR 1.38, 95% CI 1.19–1.59) but not all-cause dementia. For body size trajectories, transitioning from thinner childhood body size to overweight in adulthood was associated with lower risks of all-cause dementia (HR 0.82, 95% CI 0.75–0.90) and Alzheimer’s disease (HR 0.77, 95% CI 0.67–0.88), whereas transitioning to obesity was associated with a higher risk of vascular dementia (HR 1.54, 95% CI 1.24–1.92). In addition, higher adiposity, particularly obesity, was associated with lower gray matter and total brain volumes and greater white matter hyperintensity burden.

**Conclusion:**

Life-course adiposity patterns are associated with dementia risk and brain structural changes, with differences by subtype and sex. Childhood adiposity and weight gain may be linked to adverse outcomes, whereas moderate adiposity in adulthood was associated with lower dementia risk.

## Introduction

The global burden of dementia is rising at an alarming rate, with cases projected to exceed 150 million by 2050, posing substantial challenges for healthcare systems and aging societies (1). Despite extensive research efforts, effective disease-modifying treatments remain limited, shifting the focus toward prevention and identification of modifiable risk factors. Among these, obesity has emerged as a key candidate due to its high prevalence and its established links with cardiometabolic dysfunction (2, 3).

Several biological pathways may underlie the association between adiposity and dementia. Excess adiposity contributes to insulin resistance, chronic inflammation, and vascular dysfunction, which can promote cerebrovascular injury and increase the risk of vascular dementia. In parallel, metabolic dysregulation and inflammatory processes may also accelerate neurodegenerative changes, including amyloid deposition and neurodegeneration, linking obesity to Alzheimer’s disease (2, 3).

Adiposity, however, is a dynamic trait that evolves across the life course. While midlife obesity has been associated with increased dementia risk in several studies (4–6), evidence regarding the timing and trajectory of weight changes—and their long-term implications for brain health—remains inconsistent (7, 8). Notably, some studies have reported an inverse association between late-life overweight and dementia risk, often referred to as the “obesity paradox,” although this finding may be influenced by reverse causation and age-related changes in body composition (9). At the same time, early-life under- or over-nutrition may exert long-lasting effects on brain structure and function through developmental programming (10, 11). However, existing studies have largely examined body size at single time points, with limited investigation of how childhood body size and its transition into adulthood jointly relate to dementia risk and brain structure.

Furthermore, most previous research has relied solely on adulthood body mass index (BMI), with limited attention to longitudinal patterns or to how sex may modify these associations. Given the known sex differences in fat distribution, hormone regulation, and dementia pathophysiology, a sex-specific life-course perspective is warranted (12).

In addition to clinical outcomes, neuroimaging markers provide an opportunity to capture subclinical brain changes that may precede overt dementia, offering complementary insights into the mechanisms linking adiposity to brain health.

To address these critical gaps, we conducted a large prospective cohort study using data from the UK Biobank to investigate how childhood body size, adulthood BMI, and their combined life-course trajectories are associated with incident dementia, its subtypes (Alzheimer’s disease [AD] and vascular dementia [VD]), and neuroimaging markers of brain structure. We further explored whether these associations differed by sex.

We hypothesized that adiposity across the life course, as well as transitions between childhood and adulthood body size, are associated with dementia risk and brain structural changes, with potential differences by sex.

By integrating longitudinal adiposity measures with neurocognitive outcomes in a large, well-characterized cohort, this study aims to provide a more comprehensive understanding of the life-course relationship between body size and brain health.

## Method

### Study population

This prospective cohort study utilized data from the UK Biobank, which enrolled over 500,000 individuals aged 40–69 years for baseline assessments between 2006 and 2010 at 22 centers across England, Scotland, and Wales. Further details on the UK Biobank can be found in prior publications (13, 14).

**Figure 1:**
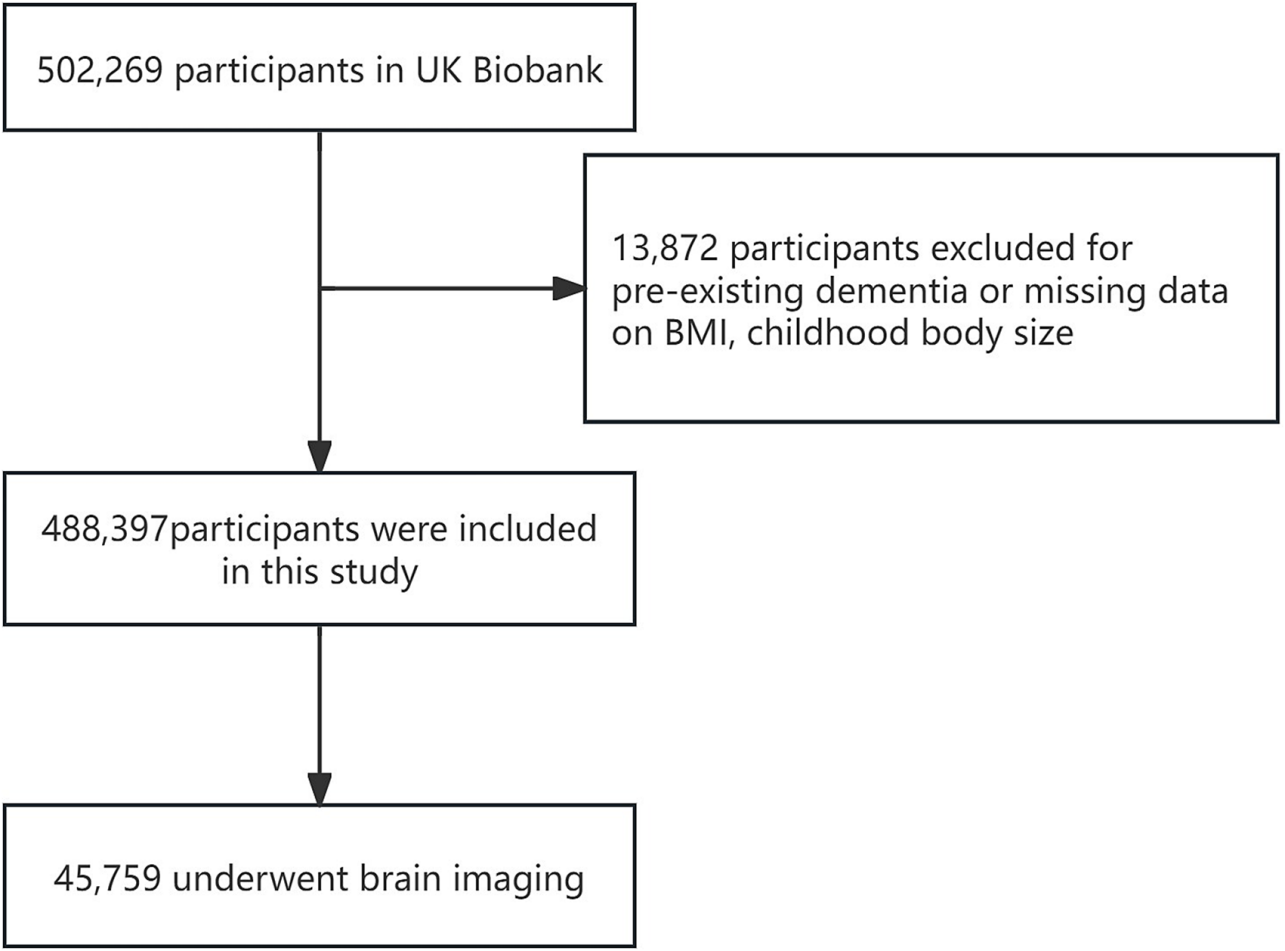
Flow chart.

For this study, participants with a baseline dementia diagnosis or missing data on height, weight, or self-reported body size at age 10 (*n* = 13,872) were excluded, resulting in a final sample of 488,397 individuals. Of these, 45,759 underwent brain imaging since 2014 and were included in a secondary analysis to examine the relationships between childhood-to-adulthood body size trajectories and brain structural measures. The inclusion and exclusion process is illustrated in Figure 1.

The study received approval from the North West Multi-Centre Research Ethics Committee (REC reference: 11/NW/03820), with all participants providing written informed consent in line with ethical standards.

### Assessment of body size in childhood and adulthood

At baseline, trained staff performed physical measurements on participants.

Body Mass Index (BMI) was calculated by dividing weight (in kilograms) by the square of height (in meters). According to World Health Organization (WHO) guidelines (15), BMI was classified into three categories: normal weight (18.5–24.9 kg/m^2^), overweight (25.0–29.9 kg/m^2^), and obesity (≥30.0 kg/m^2^).

Body fat percentage (BFP) was also assessed at baseline using bioimpedance measurements. BFP was categorized using sex-specific cut-offs as follows: in men, normal (<25%), overweight (25.0–29.9%), and obesity (≥30.0%); in women, normal (<35%), overweight (35.0–39.9%), and obesity (≥40.0%).

Childhood body size was assessed retrospectively via a self-reported question, where participants compared their body size at age 10 to that of their peers, selecting from “thinner,” “average,” or “plumper.”

To examine body size changes across the life course, we integrated childhood body size categories with adult BMI status, forming a composite variable to represent body size trajectories. This yielded nine distinct groups: average-to-normal weight, average-to-overweight, average-to-obese, thinner-to-normal weight, thinner-to-overweight, thinner-to-obese, plumper-to-normal weight, plumper-to-overweight, and plumper-to-obese. The average-to-normal weight group was used as the reference category in all analyses.

Similarly, body size trajectories were also constructed by combining childhood body size with adult BFP categories using the same classification framework.

### Outcomes ascertainment

Health outcomes were identified using hospital inpatient records sourced from the Hospital Episode Statistics (England), Scottish Morbidity Record (Scotland), and Patient Episode Database (Wales). Diagnoses were documented using the International Classification of Diseases, 10th Revision (ICD-10) coding system.

The primary outcomes of this study were dementia and its two main subtypes: Alzheimer’s disease and vascular dementia. These were defined based on ICD-10 codes as follows: dementia (F00, F01, F02, F03, F05.1, G30, G31.1, G31.8), AD (F00, G30), and VD (F01).

### Brain volume measurement

Brain imaging data were collected via magnetic resonance imaging (MRI) starting in 2014, as detailed in the UK Biobank documentation^1^. This study utilized T1-weighted MRI to measure the volumes (in cubic millimeters) of the whole brain, white matter, and gray matter. White matter hyperintensities (WMHs) volume was derived from T2-weighted MRI. To account for head size, brain tissue volumes were normalized using the estimated external skull surface from T1 images. Bilateral brain tissue volumes were summed (left and right sides) and z-standardized. Due to its skewed distribution, WMH volume was log-transformed prior to z-standardization. The primary numerical outcomes of this study included neurodegeneration-related brain volumes: whole brain volume, white matter volume, gray matter volume, and WMHs.

### Assessment of covariates

The baseline questionnaire captured data on several potential confounders, including: (1) sociodemographic factors: age, sex, ethnicity, and Townsend Deprivation Index (TDI) and educational attainment; (2) lifestyle factors: physical activity, smoking status, alcohol consumption, and dietary habits (vegetable, fruit, and processed meat intake).

Smoking and alcohol behaviors were classified as never, former, or current. Socioeconomic status was assessed using the TDI, calculated from participants’ residential postcodes and national census data, with higher scores reflecting greater deprivation (16). Self-reported ethnicity was grouped into four categories: White, Black, Asian, and Other. Sleep duration was self-reported and divided into short (<7 h), normal (7–9 h), or long (>9 h). Physical activity was quantified as total metabolic equivalent task (MET) hours per week, encompassing walking, moderate, and vigorous activities. Vegetable and fruit consumption were measured as the median daily intake of heaped tablespoons of vegetables and pieces of fruit, respectively. Processed meat consumption was categorized as never, less than once weekly, once weekly, or more than twice weekly.

### Statistical analyses

Baseline characteristics were summarized using descriptive statistics. Continuous variables were reported as means with standard deviations (SDs) for normally distributed data or as medians with interquartile ranges (IQRs) for non-normally distributed data. Categorical variables were presented as counts and percentages. Cox proportional hazards regression models were used to assess the relationships between childhood body size, adult BMI, and body size trajectories from childhood to adulthood with the risk of incident all-cause dementia. Results were reported as hazard ratios (HRs) with 95% confidence intervals (CIs).

All models were adjusted for confounders, including age, sex, Townsend Deprivation Index (TDI), educational attainment, smoking status, alcohol consumption, ethnicity, sleep duration, total physical activity, and intake of fruits, vegetables, and processed meat.

To evaluate the robustness of the findings, several sensitivity analyses were conducted: (1) excluding incident cases within the first 2 years of follow-up to minimize reverse causation; (2) additionally excluding cases occurring within the first 5 years of follow-up to further address potential reverse causation; (3) using multiple imputation to handle missing covariate data; (4) excluding participants with missing covariate data; and (5) performing sex-stratified analyses (women vs. men) to investigate potential sex-based effect modification, and (6) applying competing risk models to account for the potential influence of death as a competing event.

In addition, exploratory analyses were conducted by replacing BMI with BFP to assess the robustness of the associations using an alternative adiposity measure.

Statistical analyses were performed using R software (version 4.3.1). Two-sided *p*-values were reported, with statistical significance set at *p* < 0.05.

## Results

### Baseline characteristics

The study cohort included 488,397 participants, with a median age of 58.00 years (IQR: 50.00–63.00). Females comprised 54.6% of the cohort, and males 45.4%. Adulthood BMI distribution showed 33.1% normal weight, 41.9% overweight, and 25.0% obese. Childhood body size was reported as thinner (33.3%), average (50.8%), or plumper (15.9%). The most common body size trajectory was average childhood to overweight adulthood (22.3%), followed by average to normal weight (16.7%) and average to obese (11.8%) (Table 1).

**Table 1:** Baseline characteristics of the study population.

Characteristics	All
Sample size	488,397
Age [median (IQR)]	58.00 [50.00, 63.00]
Sex	
Female	266,807 (54.6)
Male	221,590 (45.4)
Townsend deprivation index [median (IQR)]	−2.17 [−3.66, 0.49]
Smoking (%)	
Never	266,256 (54.7)
Former	169,336 (34.8)
Current	51,088 (10.5)
Alcohol status	
Never	20,867 (4.3)
Former	17,437 (3.6)
Current	449,616 (92.1)
Ethnicity	
White	466,354 (95.5)
Black	11,173 (2.3)
Asian	3,151 (0.6)
Other	7,719 (1.6)
Sleep time	
Short	119,521 (24.6)
Normal	357,381 (73.6)
Long	8,848 (1.8)
Adulthood BMI (kg/m^2^)	
Normal weight	161,706 (33.1)
Overweight	204,621 (41.9)
Obesity	122,070 (25.0)
Childhood body size	
Thinner	162,612 (33.3)
Average	248,133 (50.8)
Plumper	77,652 (15.9)
Body size change	
Thinner → normal weight	65,256 (13.4)
Thinner → overweight	66,128 (13.5)
Thinner → obesity	31,228 (6.4)
Average → normal weight	81,564 (16.7)
Average → overweight	108,710 (22.3)
Average → obesity	57,859 (11.8)
Plumper → normal weight	14,886 (3.0)
Plumper → overweight	29,783 (6.1)
Plumper → obesity	32,983 (6.8)
Education, years	12.00 [10.00, 20.00]
Fitness and lifestyle	
Total physical activity, MET-h per week	29.77 [13.62, 59.50]
Fresh fruit intake, median (IQR), pieces/day	2.00 [1.00, 3.00]
Vegetable intake, median (IQR), tablespoons/day	4.00 [3.00, 6.00]
Processed meat intake, *n* (%)	
Never	45,330 (9.3)
Less than once a week	148,754 (30.5)
Once a week	142,546 (29.2)
More than twice a week	150,866 (30.9)

### Body size and risk of dementia

Plumper body size in childhood was associated with a higher risk of all-cause dementia compared with average childhood body size (HR 1.12, 95% CI 1.04–1.20), while no significant associations were observed for AD or VD. Thinner childhood body size was not associated with dementia outcomes.

In adulthood, overweight was associated with lower risks of all-cause dementia (HR 0.86, 95% CI 0.81–0.92) and AD (HR 0.83, 95% CI 0.76–0.91), but not VD. Obesity was associated with lower risk of AD (HR 0.82, 95% CI 0.74–0.91) and higher risk of VD (HR 1.38, 95% CI 1.19–1.59), while no significant association was observed for all-cause dementia.

**Figure 2:**
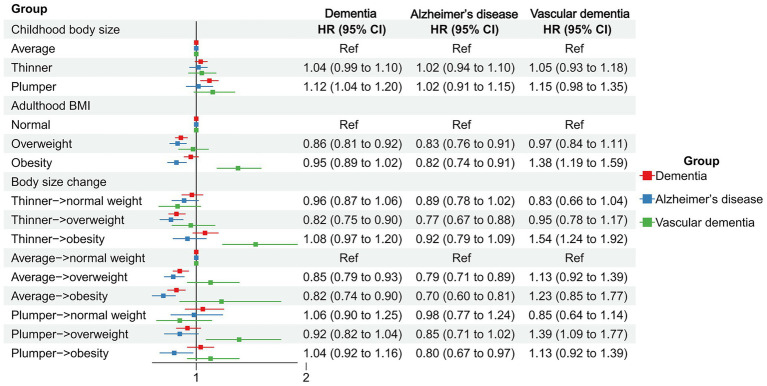
Association of childhood body size, adulthood BMI, body size change with Dementia.

When examining body size trajectories, compared with the reference group (average childhood body size to normal weight in adulthood), transitioning from thinner to overweight was associated with lower risks of all-cause dementia (HR 0.82, 95% CI 0.75–0.90) and AD (HR 0.77, 95% CI 0.67–0.88), whereas transitioning from thinner to obesity was associated with a higher risk of VD (HR 1.54, 95% CI 1.24–1.92). Transitions from average childhood body size to overweight or obesity were both associated with lower risks of all-cause dementia and AD, with no significant associations observed for VD. Among individuals with plumper childhood body size, transitioning to overweight was associated with higher risk of VD (HR 1.39, 95% CI 1.09–1.77), while transitioning to obesity was associated with lower risk of AD (HR 0.80, 95% CI 0.67–0.97) (Figure 2).

### Body size and brain structure

Thinner childhood body size was associated with higher gray matter, white matter, and total brain volumes, with no significant association observed for WMH. In contrast, plumper childhood body size was associated with lower gray matter and total brain volumes.

In adulthood, overweight was associated with lower gray matter and total brain volumes and higher WMH volume, while obesity was associated with lower gray matter and total brain volumes and higher white matter and WMH volume.

**Figure 3:**
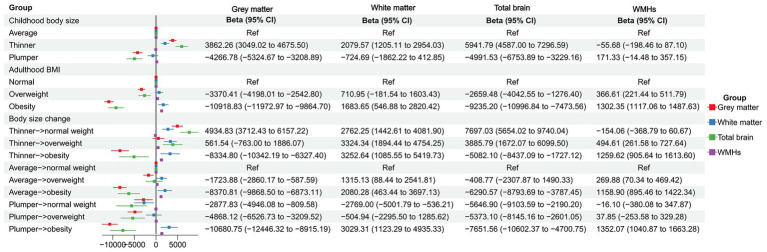
Association of childhood body size, adulthood BMI, body size change with brain structure.

For body size trajectories, transitioning from thinner to normal weight was associated with higher gray matter, white matter, and total brain volumes, while transitioning from thinner to overweight was associated with higher white matter and total brain volumes and higher WMH volume. Transitioning from thinner to obesity was associated with lower gray matter and total brain volumes and higher white matter and WMH volume. Transitions from average childhood body size to overweight or obesity were associated with lower gray matter and higher WMH volume, with obesity also associated with lower total brain volume. Among those with plumper childhood body size, most trajectories were associated with lower gray matter and total brain volumes, while transitioning to obesity was additionally associated with higher white matter and WMH volume (Figure 3).

### Sex-stratified analyses

In sex-stratified analyses, plumper childhood body size was associated with higher risk of all-cause dementia in men (HR 1.15, 95% CI 1.04–1.28), but not in women. Overweight in adulthood was associated with lower risks of all-cause dementia and AD in both sexes. Obesity was associated with lower AD risk in women (HR 0.75, 95% CI 0.65–0.87) and higher VD risk in men (HR 1.44, 95% CI 1.19–1.74) (Supplementary Table S1).

For body size trajectories, transitions to overweight were associated with lower risks of dementia and AD, particularly in men, whereas transitions to obesity were associated with higher VD risk in both sexes, including thinner-to-obesity and plumper-to-obesity (Supplementary Table S1). For brain structure, obesity was associated with lower gray matter and higher WMH volume in both sexes, with larger effect sizes observed in men (Supplementary Tables S2, S3).

### Sensitivity analysis

The associations of childhood body size, adulthood BMI, and body size change with incident dementia outcomes remained largely consistent across multiple sensitivity analyses: when excluding incident cases that occurred within the first 2 years of follow-up (Supplementary Table S4); when further excluding cases within the first 5 years (Supplementary Table S5); when restricting analyses to participants with complete covariate data (Supplementary Table S6); and when using multiple imputation for missing covariates (Supplementary Table S7). Similar patterns were also observed in competing risk models (Supplementary Table S8).

### Exploratory analysis

Analyses using BFP yielded similar patterns (Supplementary Tables S9, S10). Overweight BFP was associated with lower risks of all-cause dementia (HR 0.91, 95% CI 0.86–0.97) and AD (HR 0.86, 95% CI 0.78–0.94), while obesity was associated with lower AD risk (HR 0.84, 95% CI 0.77–0.93) and higher VD risk (HR 1.26, 95% CI 1.10–1.45) (Supplementary Table S9). For body size trajectories, transitions to overweight were associated with lower risks of dementia and AD, whereas transitions to obesity were associated with higher VD risk (Supplementary Table S9). Higher BFP was also associated with lower gray matter and total brain volumes and higher WMH volume, with stronger associations observed for obesity (Supplementary Table S10).

## Discussion

This large, population-based cohort study suggests that both childhood and adulthood adiposity, as well as their longitudinal interplay, are associated with the risk of dementia and alterations in brain structure. Leveraging comprehensive phenotyping and imaging from the UK Biobank, our findings contribute novel insights into how life-course body size trajectories shape late-life neurocognitive outcomes. Notably, distinct patterns emerged for AD and VD, and sex-specific differences highlighted the need for tailored preventive strategies. Importantly, similar patterns were observed in exploratory analyses using BFP and in competing risk models, further supporting the robustness of these findings across different adiposity measures and analytic approaches.

We observed that individuals with a plumper body size in childhood had an elevated risk of all-cause dementia, with more consistent associations seen in males. Furthermore, this group exhibited reduced gray matter and total brain volumes in later life. These findings align with the developmental origins of health and disease (DOHaD) hypothesis, which postulates that early-life nutritional and metabolic insults may be associated with enduring physiological alterations (17, 18). Childhood obesity is associated with systemic inflammation, altered hypothalamic–pituitary–adrenal (HPA) axis function, and insulin resistance—all of which may negatively influence brain development (19). Reduced brain volumes observed in this group may reflect disrupted neurogenesis and synaptic pruning during critical periods of brain maturation (20). Additionally, early-life adiposity may be linked to long-term metabolic and vascular alterations, potentially increasing susceptibility to later-life neurodegeneration.

Interestingly, thinner childhood body size was not significantly associated with dementia outcomes, suggesting a more limited or context-dependent role. This finding indicates that early-life leanness alone may not substantially influence dementia risk in later life, although its impact may depend on subsequent life-course weight trajectories and environmental exposures.

Adulthood overweight was paradoxically associated with a reduced risk of all-cause dementia and AD, particularly among men. This so-called “obesity paradox” in cognitive aging may partly explain these findings, although alternative explanations such as reverse causation should be considered. Higher BMI in later adulthood may reflect differences in nutritional status or body composition; however, these explanations remain speculative (3). Additionally, reverse causality may be at play, as preclinical dementia is often associated with weight loss, which could inflate the apparent protective effects of higher BMI (21). Another possible explanation is that moderate adiposity may provide metabolic reserves during catabolic states associated with aging and neurodegeneration (22, 23). Notably, similar patterns were observed when using BFP as an alternative adiposity indicator, with overweight BFP showing protective associations for all-cause dementia and AD. Notably, despite these protective associations for clinical outcomes, overweight was also associated with less favorable brain structural profiles, suggesting that clinical and subclinical markers may not always align and may reflect different underlying mechanisms or time scales.

In contrast, obesity—especially when persisting from childhood or resulting from substantial weight gain—was associated with a higher risk of VD and adverse brain structural changes, such as increased WMHs and reduced gray matter volume. These structural markers are indicative of small vessel disease, which may be related to endothelial dysfunction associated with obesity, hypertension, and chronic inflammation (24). WMHs have been consistently associated with executive dysfunction and gait disturbances, hallmarks of vascular cognitive impairment (25). This pattern was also observed in BFP-based analyses, where obesity was associated with increased VD risk and greater WMH burden. Excess adiposity may further exacerbate cerebrovascular damage through impaired cerebral perfusion and blood–brain barrier disruption.

Our investigation into life-course body size changes revealed nuanced and biologically plausible patterns. Individuals who were thin in childhood but became overweight—not obese—in adulthood had a lower risk of dementia and demonstrated increased white and total brain volumes. This suggests that moderate weight gain from a lean baseline may reflect improved socioeconomic status, better nutrition, and enhanced brain reserve. Consistent patterns were also observed in analyses based on BFP-defined trajectories.

Conversely, transitions to obesity—particularly from thinner or plumper childhood body size—were consistently associated with increased risk of VD, smaller gray matter volumes, and greater WMH burden. These findings suggest links between excessive weight gain and metabolic, vascular, and inflammatory pathways. Notably, this pattern held across both sexes, although the magnitude and affected brain regions differed somewhat. The consistency of these findings across BMI- and BFP-based analyses further strengthens the robustness of this observation. These results support the importance of cumulative adiposity burden across the life course.

The stratified analyses revealed important sex-based differences in the adiposity-dementia relationship. Among men, plumper childhood body size was a stronger predictor of later dementia, suggesting potential sex-specific differences in susceptibility. Adulthood overweight was more consistently associated with lower dementia risk, whereas obesity-related VD risk appeared more pronounced in men. These differences may be related to sex-specific fat distribution patterns and hormonal factors, including visceral adiposity and estrogen exposure (26, 27).

Several interrelated biological pathways may help explain the observed associations between life-course adiposity patterns and late-life brain health. These may include chronic low-grade systemic inflammation, insulin resistance, and vascular dysfunction, which are closely linked to adiposity (28). Adipose tissue secretes pro-inflammatory cytokines such as interleukin-6 (IL-6), tumor necrosis factor-alpha (TNF-α), and C-reactive protein (CRP), which may cross the blood–brain barrier and contribute to neuroinflammatory processes (29, 30). Insulin resistance and vascular dysfunction may contribute to both neurodegenerative and cerebrovascular processes (31, 32).

Vascular mechanisms also play a prominent role, particularly in the context of vascular dementia and WMH accumulation. Excess body weight is associated with endothelial dysfunction, arterial stiffness, and increased risk of hypertension and atherosclerosis, all of which contribute to cerebral small vessel disease (33, 34). These vascular changes can reduce perfusion, compromise BBB integrity, and promote ischemic injury in the deep white matter regions of the brain, which manifests as WMHs on imaging and is strongly linked to executive dysfunction and cognitive impairment (35).

Moreover, adiposity may influence neuroplasticity and brain structural integrity via alterations in adipokines. Hormones such as leptin and adiponectin, which are dysregulated in obesity, have important roles in modulating synaptic activity, neurogenesis, and hippocampal function (36). Leptin resistance, for example, may blunt its neuroprotective effects, while low adiponectin levels are associated with impaired endothelial function and greater neuroinflammation (30, 36). Obesity-related oxidative stress and mitochondrial dysfunction further impair neural homeostasis and may accelerate age-related neurodegenerative changes (30, 31). Finally, early-life adiposity may disrupt key developmental processes including synaptic pruning, myelination, and white matter tract maturation, leading to persistent structural vulnerabilities that predispose individuals to cognitive decline decades later (20, 32).

Our findings highlight the critical importance of adopting a life-course approach to dementia prevention. Strategies that promote optimal growth in childhood—avoiding both under- and over-nutrition—along with maintenance of a healthy weight in adulthood may substantially reduce dementia burden. Recognizing that moderate overweight in later life may not be uniformly harmful challenges simplistic BMI-based risk categorizations and emphasizes the need for a more nuanced assessment that includes body composition, fitness, and metabolic health. The consistency between BMI- and BFP-based findings further supports the value of incorporating alternative adiposity measures in epidemiological studies.

Moreover, our study supports the integration of brain imaging markers such as WMHs and gray matter volume in obesity research. These neuroimaging phenotypes may serve as intermediate biomarkers for identifying high-risk individuals and evaluating intervention efficacy before clinical symptoms appear.

Building upon these findings, several important avenues for future research emerge. First, studies incorporating longitudinal assessments of circulating biomarkers—such as inflammatory cytokines, insulin sensitivity indices, neurotrophic factors (e.g., brain-derived neurotrophic factor), and lipid metabolites—are needed to elucidate the temporal sequence and biological pathways linking adiposity to neurocognitive outcomes. Such work may benefit from integrative multi-omics approaches, including genomics, epigenetics, transcriptomics, and metabolomics, to uncover novel mechanistic insights and therapeutic targets.

Second, interventional studies are warranted to determine whether modifying adiposity trajectories at specific life stages can influence brain aging and dementia risk. Trials targeting early-life nutrition, weight stabilization during adolescence, or midlife metabolic control may clarify the critical windows for effective prevention. Lifestyle interventions that combine physical activity, dietary optimization, and cognitive training should be evaluated not only for weight outcomes but also for effects on neuroimaging markers, cognitive performance, and clinical dementia incidence.

Third, expanding these investigations into more ethnically and socioeconomically diverse populations is essential to ensure generalizability and address disparities in both obesity and dementia burden. The UK Biobank cohort, while rich in data, underrepresents non-White populations, limiting external validity. Future large-scale, multiethnic cohorts will be critical in confirming and extending these findings.

Finally, predictive modeling efforts should integrate body size trajectories with other established dementia risk factors—including genetic predisposition (e.g., APOE ε4), cardiovascular risk scores, education, and cognitive reserve—to develop individualized risk stratification tools. These models can inform precision prevention strategies and guide the timing and content of targeted interventions across the lifespan.

Key strengths of our study include its large sample size, prospective design, rich phenotypic data, and neuroimaging endpoints. The availability of body size data across the life course allowed us to model dynamic changes rather than rely on static measures.

However, several limitations should be acknowledged. First, childhood body size was retrospectively self-reported using subjective categories, which may be prone to recall bias and misclassification. Although this measure lacks direct validation within the UK Biobank, it has been widely used in prior studies (37, 38). Second, BMI is an imperfect proxy for adiposity as it does not distinguish between fat and lean mass. While this was partly addressed using BFP in sensitivity analyses, more precise body composition measures are needed, and future studies using DXA or MRI may provide more accurate characterization of fat distribution and its neurological relevance. Third, residual confounding cannot be excluded despite extensive adjustment. In particular, unmeasured or imperfectly measured factors, including lifestyle, metabolic status, and early-life environment, may have influenced the results. In addition, dietary assessment was limited, and total energy intake was unavailable, which may further contribute to residual confounding. Finally, the UK Biobank cohort is predominantly of White European ancestry, which may limit generalizability to more diverse populations.

## Conclusion

In conclusion, body size across the life course is associated with dementia risk and brain structural changes. These patterns were consistent across BMI- and body fat percentage–based measures. Moderate adiposity in adulthood was associated with lower risks of all-cause dementia and Alzheimer’s disease, whereas obesity—particularly across the life course—was associated with higher vascular dementia risk and adverse brain structural profiles. A life-course perspective on adiposity may improve risk stratification and inform dementia prevention strategies.

## Data Availability

Publicly available datasets were analyzed in this study. This data can be found: https://www.ukbiobank.ac.uk.

## Footnotes

1
https://biobank.ndph.ox.ac.uk/showcase/showcase/docs/brain_mri.pdf
