# Supplementary material for: Associations of childhood-to-adulthood body size trajectories with dementia risk and brain structural imaging markers: a study from UK Biobank
Source: Front Nutr. 2026 Jul 28;13:1764658. doi: 10.3389/fnut.2026.1764658 (PMC13457295; doi:10.3389/fnut.2026.1764658)
Supplement: Supplementary file 1 [file Table_1.DOCX]

Table S1. Association of childhood body size, adulthood BMI, body size change with Dementia stratified by sex

|  | Women | | | | | | Men | | | | | |
| --- | --- | --- | --- | --- | --- | --- | --- | --- | --- | --- | --- | --- |
|  | Dementia |  | AD |  | VD |  | Dementia |  | AD |  | VD |  |
|  | HR (95%CI) | P | HR (95%CI) | P | HR (95%CI) | P | HR (95%CI) | P | HR (95%CI) | P | HR (95%CI) | P |
| Childhood body size |  |  |  |  |  |  |  |  |  |  |  |  |
| Average | 1(Ref) |  | 1(Ref) |  | 1(Ref) |  | 1(Ref) |  | 1(Ref) |  | 1(Ref) |  |
| Thinner | 1.1(1.01 - 1.2) | 0.024 | 1.08(0.96 - 1.22) | 0.195 | 1.1(0.91 - 1.34) | 0.338 | 1.01(0.94 - 1.09) | 0.741 | 0.98(0.87 - 1.09) | 0.686 | 1.04(0.9 - 1.21) | 0.57 |
| Plumper | 1.08(0.97 - 1.21) | 0.142 | 1(0.86 - 1.17) | 0.978 | 1.15(0.9 - 1.46) | 0.28 | 1.15(1.04 - 1.27) | 0.007 | 1.05(0.89 - 1.24) | 0.553 | 1.16(0.94 - 1.42) | 0.169 |
| Adulthood BMI |  |  |  |  |  |  |  |  |  |  |  |  |
| Normal | 1(Ref) |  | 1(Ref) |  | 1(Ref) |  | 1(Ref) |  | 1(Ref) |  | 1(Ref) |  |
| Overweight | 0.93(0.85 - 1.01) | 0.086 | 0.85(0.75 - 0.96) | 0.008 | 1.09(0.89 - 1.35) | 0.401 | 0.86(0.79 - 0.93) | 0 | 0.87(0.77 - 0.99) | 0.031 | 0.96(0.8 - 1.15) | 0.636 |
| Obesity | 0.96(0.86 - 1.06) | 0.381 | 0.79(0.68 - 0.91) | 0.002 | 1.32(1.05 - 1.66) | 0.016 | 1.03(0.94 - 1.13) | 0.497 | 0.95(0.82 - 1.1) | 0.475 | 1.56(1.29 - 1.88) | <0.001 |
| Body size change |  |  |  |  |  |  |  |  |  |  |  |  |
| Thinner→normal weight | 0.93(0.81 - 1.06) | 0.257 | 0.89(0.74 - 1.07) | 0.204 | 0.95(0.68 - 1.34) | 0.781 | 1(0.87 - 1.14) | 0.966 | 0.91(0.74 - 1.12) | 0.372 | 0.74(0.54 - 1.01) | 0.055 |
| Thinner→overweight | 0.95(0.84 - 1.09) | 0.49 | 0.87(0.72 - 1.04) | 0.126 | 1.18(0.86 - 1.62) | 0.313 | 0.8(0.71 - 0.91) | 0.001 | 0.77(0.63 - 0.93) | 0.008 | 0.9(0.69 - 1.17) | 0.417 |
| Thinner→obesity | 1.15(0.98 - 1.35) | 0.081 | 0.97(0.78 - 1.22) | 0.807 | 1.59(1.12 - 2.27) | 0.01 | 1.17(1.01 - 1.36) | 0.035 | 1.03(0.82 - 1.3) | 0.808 | 1.7(1.28 - 2.24) | <0.001 |
| Average→normal weight | 1(Ref) |  | 1(Ref) |  | 1(Ref) |  | 1(Ref) |  | 1(Ref) |  | 1(Ref) |  |
| Average→overweight | 0.86(0.76 - 0.96) | 0.01 | 0.77(0.66 - 0.91) | 0.002 | 1.06(0.8 - 1.41) | 0.686 | 0.89(0.8 - 1) | 0.05 | 0.86(0.72 - 1.02) | 0.083 | 0.85(0.67 - 1.09) | 0.195 |
| Average→obesity | 0.77(0.66 - 0.9) | 0.001 | 0.63(0.51 - 0.79) | <0.001 | 1.2(0.86 - 1.68) | 0.272 | 0.92(0.8 - 1.05) | 0.208 | 0.83(0.67 - 1.02) | 0.07 | 1.17(0.9 - 1.52) | 0.244 |
| Plumper→normal weight | 0.99(0.8 - 1.21) | 0.892 | 0.96(0.72 - 1.28) | 0.79 | 1.29(0.8 - 2.07) | 0.3 | 1.16(0.88 - 1.52) | 0.291 | 0.95(0.61 - 1.49) | 0.824 | 1.15(0.65 - 2.06) | 0.629 |
| Plumper→overweight | 0.95(0.8 - 1.13) | 0.541 | 0.83(0.65 - 1.07) | 0.152 | 1.12(0.74 - 1.69) | 0.606 | 0.94(0.79 - 1.11) | 0.443 | 0.91(0.7 - 1.18) | 0.46 | 0.71(0.48 - 1.06) | 0.093 |
| Plumper→obesity | 0.98(0.82 - 1.16) | 0.784 | 0.75(0.57 - 0.97) | 0.027 | 1.3(0.88 - 1.93) | 0.192 | 1.17(0.99 - 1.37) | 0.059 | 0.95(0.73 - 1.23) | 0.677 | 1.53(1.12 - 2.09) | 0.007 |

Table S2. Association of childhood body size, adulthood BMI, body size change with brain structure in women

|  | Grey matter | | White matter | | Total brain | | Volume of white matter hyperintensities | |
| --- | --- | --- | --- | --- | --- | --- | --- | --- |
|  | β coefcients  (95%CI) | P | β coefcients  (95%CI) | P | β coefcients  (95%CI) | P | β coefcients  (95%CI) | P |
| Childhood body size |  |  |  |  |  |  |  |  |
| Average | 1(Ref) |  | 1(Ref) |  | 1(Ref) |  | 1(Ref) |  |
| Thinner | 4205.45(3052 to 5358.9) | <0.001 | 1778.49(545.19 to 3011.79) | 0.005 | 5983.92(4056.94 to 7910.9) | <0.001 | -74.08(-262.87 to 114.7) | 0.442 |
| Plumper | -3544.84(-4950.88 to -2138.79) | <0.001 | -361.49(-1864.87 to 1141.89) | 0.637 | -3906.33(-6255.29 to -1557.36) | 0.001 | 95.85(-134.49 to 326.19) | 0.415 |
| Adulthood BMI |  |  |  |  |  |  |  |  |
| Normal | 1(Ref) |  | 1(Ref) |  | 1(Ref) |  | 1(Ref) |  |
| Overweight | -1144.63(-2284.22 to -5.05) | 0.049 | 1604.31(387.97 to 2820.66) | 0.01 | 459.68(-1445.13 to 2364.48) | 0.636 | 239.76(53.91 to 425.62) | 0.011 |
| Obesity | -5836.5(-7282.21 to -4390.8) | <0.001 | 4547.74(3004.66 to 6090.83) | <0.001 | -1288.81(-3705.29 to 1127.68) | 0.296 | 828.83(592.4 to 1065.27) | <0.001 |
| Body size change |  |  |  |  |  |  |  |  |
| Thinner→normal weight | 4823.17(3262.99 to 6383.36) | <0.001 | 2014.51(346.98 to 3682.05) | 0.018 | 6837.68(4229.17 to 9446.2) | <0.001 | -195.6(-450.76 to 59.57) | 0.133 |
| Thinner→overweight | 3574.51(1642.33 to 5506.69) | <0.001 | 4227.63(2162.51 to 6292.75) | <0.001 | 7802.09(4571.63 to 11032.54) | <0.001 | 319.36(2.72 to 636) | 0.048 |
| Thinner→obesity | -2366.34(-5275.97 to 543.29) | 0.111 | 5193.62(2083.79 to 8303.45) | 0.001 | 2827.24(-2037.45 to 7691.92) | 0.255 | 663.9(186.42 to 1141.38) | 0.006 |
| Average→normal weight | 1(Ref) |  | 1(Ref) |  | 1(Ref) |  | 1(Ref) |  |
| Average→overweight | -62.34(-1617.12 to 1492.45) | 0.937 | 2046.75(384.99 to 3708.51) | 0.016 | 1984.42(-615.07 to 4583.9) | 0.135 | 123.34(-130.51 to 377.2) | 0.341 |
| Average→obesity | -3595.4(-5672.78to-1518.02) | 0.001 | 3587.98(1367.66to5808.3) | 0.002 | -7.46(-3480.68to3465.77) | 0.997 | 576.04(236.66to915.42) | 0.001 |
| Plumper→normal weight | -2685.3(-5113.97to-256.63) | 0.03 | -2898.31(-5494.08to-302.53) | 0.029 | -5583.6(-9644.15to-1523.05) | 0.007 | -235.06(-632.59to162.46) | 0.246 |
| Plumper→overweight | -3180.4(-5411.79to-949.01) | 0.005 | -1178.88(-3563.8to1206.04) | 0.333 | -4359.25(-8089.96to-628.53) | 0.022 | -57.48(-422.01to307.05) | 0.757 |
| Plumper→obesity | -6521.65(-8873.21to-4170.09) | <0.001 | 6786.39(4273.02to9299.75) | <0.001 | 264.67(-3666.96to4196.3) | 0.895 | 990.5(603.46to1377.54) | <0.001 |

Table S3. Association of childhood body size, adulthood BMI, body size change with brain structure in men

|  | Grey matter | | White matter | | Total brain | | Volume of white matter hyperintensities | |
| --- | --- | --- | --- | --- | --- | --- | --- | --- |
|  | β coefcients  (95%CI) | P | β coefcients  (95%CI) | P | β coefcients  (95%CI) | P | β coefcients  (95%CI) | P |
| Childhood body size |  |  |  |  |  |  |  |  |
| Average | 1(Ref) |  | 1(Ref) |  | 1(Ref) |  | 1(Ref) |  |
| Thinner | 3593.49(2447.21 to 4739.76) | <0.001 | 2394.45(1152.83 to 3636.08) | <0.001 | 5987.88(4082.69 to 7893.06) | <0.001 | -39.81(-254.45 to 174.82) | 0.716 |
| Plumper | -5324.47(-6927.06 to -3721.87) | <0.001 | -1392.15(-3128.05 to 343.75) | 0.116 | -6716.74(-9380.35 to -4053.13) | <0.001 | 261.53(-38.57 to 561.62) | 0.088 |
| Adulthood BMI |  |  |  |  |  |  |  |  |
| Normal | 1(Ref) |  | 1(Ref) |  | 1(Ref) |  | 1(Ref) |  |
| Overweight | -5520.61(-6727.46 to -4313.77) | <0.001 | 29.71(-1287.59 to 1347) | 0.965 | -5490.93(-7508 to -3473.86) | <0.001 | 573.64(347.26 to 800.03) | <0.001 |
| Obesity | -15248.87(-16783.53 to -13714.22) | <0.001 | -429.95(-2105.06 to 1245.15) | 0.615 | -15678.81(-18243.77 to -13113.86) | <0.001 | 1818.63(1530.34 to 2106.92) | <0.001 |
| Body size change |  |  |  |  |  |  |  |  |
| Thinner→normal weight | 4020.16(2042.54 to 5997.77) | <0.001 | 3408.36(1248.53 to 5568.18) | 0.002 | 7428.38(4123.59 to 10733.16) | <0.001 | -1.23(-372.4 to 369.94) | 0.995 |
| Thinner→overweight | -2148.08(-4066.11 to -230.05) | 0.028 | 2992.36(897.61 to 5087.11) | 0.005 | 844.12(-2361.09 to 4049.34) | 0.606 | 804.29(444.04 to 1164.53) | <0.001 |
| Thinner→obesity | -12641.01(-15459.59 to -9822.43) | <0.001 | 2724.27(-354.01 to 5802.54) | 0.083 | -9916.64(-14626.77 to -5206.52) | <0.001 | 1895.85(1364.8 to 2426.9) | <0.001 |
| Average→normal weight | 1(Ref) |  | 1(Ref) |  | 1(Ref) |  | 1(Ref) |  |
| Average→overweight | -3932.1(-5664.52 to -2199.67) | <0.001 | 879.42(-1012.63 to 2771.46) | 0.362 | -3052.75(-5947.79 to -157.7) | 0.039 | 563.87(238.68 to 889.06) | 0.001 |
| Average→obesity | -12737.68(-14947.74to-10527.63) | <0.001 | 1301.67(-1112.01to3715.35) | 0.29 | -11436.1(-15129.31to-7742.88) | <0.001 | 1820.89(1404.97to2236.8) | <0.001 |
| Plumper→normal weight | -3193.92(-7124.44to736.61) | 0.111 | -2895.9(-7188.57to1396.77) | 0.186 | -6090.06(-12658.35to478.22) | 0.069 | 490.74(-250.68to1232.16) | 0.195 |
| Plumper→overweight | -7428.21(-9939.15to-4917.26) | <0.001 | 247.65(-2494.65to2989.94) | 0.86 | -7180.7(-11376.73to-2984.66) | 0.001 | 275.22(-196.69to747.13) | 0.253 |
| Plumper→obesity | -15657.38(-18351.25to-12963.51) | <0.001 | -902.46(-3844.53to2039.62) | 0.548 | -16560.04(-21061.76to-12058.32) | <0.001 | 1870.72(1365.18to2376.25) | <0.001 |

Table S4. Association of childhood body size, adulthood BMI, body size change with Dementia excluding the first two years.

|  | Dementia |  | AD |  | VD |  |
| --- | --- | --- | --- | --- | --- | --- |
|  | HR (95%CI) | P | HR (95%CI) | P | HR (95%CI) | P |
| Childhood body size |  |  |  |  |  |  |
| Average | 1(Ref) |  | 1(Ref) |  | 1(Ref) |  |
| Thinner | 1.05(0.99 - 1.11) | 0.096 | 1.02(0.94 - 1.11) | 0.593 | 1.07(0.95 - 1.2) | 0.284 |
| Plumper | 1.11(1.03 - 1.2) | 0.006 | 1.02(0.91 - 1.14) | 0.708 | 1.13(0.96 - 1.33) | 0.128 |
| Adulthood BMI |  |  |  |  |  |  |
| Normal | 1(Ref) |  | 1(Ref) |  | 1(Ref) |  |
| Overweight | 0.89(0.83 - 0.94) | <0.001 | 0.85(0.78 - 0.93) | <0.001 | 1.01(0.88 - 1.16) | 0.914 |
| Obesity | 1.01(0.94 - 1.08) | 0.88 | 0.87(0.79 - 0.97) | 0.01 | 1.48(1.28 - 1.71) | <0.001 |
| Body size change |  |  |  |  |  |  |
| Thinner→normal weight | 0.95(0.87 - 1.05) | 0.33 | 0.88(0.77 - 1.01) | 0.079 | 0.82(0.65 - 1.03) | 0.084 |
| Thinner→overweight | 0.85(0.78 - 0.93) | 0.001 | 0.8(0.7 - 0.91) | 0.001 | 1.01(0.82 - 1.23) | 0.935 |
| Thinner→obesity | 1.16(1.04 - 1.29) | 0.007 | 1(0.85 - 1.17) | 0.991 | 1.69(1.36 - 2.1) | <0.001 |
| Average→normal weight | 1(Ref) |  | 1(Ref) |  | 1(Ref) |  |
| Average→overweight | 0.87(0.8 - 0.94) | 0.001 | 0.81(0.72 - 0.91) | <0.001 | 0.92(0.77 - 1.11) | 0.405 |
| Average→obesity | 0.86(0.78 - 0.95) | 0.002 | 0.73(0.63 - 0.85) | <0.001 | 1.21(0.98 - 1.48) | 0.072 |
| Plumper→normal weight | 1.04(0.88 - 1.23) | 0.633 | 0.97(0.76 - 1.23) | 0.801 | 1.18(0.81 - 1.71) | 0.382 |
| Plumper→overweight | 0.93(0.83 - 1.05) | 0.261 | 0.86(0.72 - 1.03) | 0.097 | 0.86(0.65 - 1.15) | 0.32 |
| Plumper→obesity | 1.07(0.95 - 1.2) | 0.275 | 0.84(0.7 - 1.01) | 0.058 | 1.43(1.12 - 1.83) | 0.004 |

Table S5. Association of childhood body size, adulthood BMI, body size change with Dementia by excluding covariate missing values.

|  | Dementia |  | AD |  | VD |  |
| --- | --- | --- | --- | --- | --- | --- |
|  | HR (95%CI) | P | HR (95%CI) | P | HR (95%CI) | P |
| Childhood body size |  |  |  |  |  |  |
| Average | 1(Ref) |  | 1(Ref) |  | 1(Ref) |  |
| Thinner | 1.05(0.99 - 1.11) | 0.079 | 1.03(0.95 - 1.11) | 0.539 | 1.07(0.95 - 1.2) | 0.287 |
| Plumper | 1.12(1.04 - 1.2) | 0.004 | 1.02(0.91 - 1.14) | 0.7 | 1.15(0.98 - 1.35) | 0.086 |
| Adulthood BMI |  |  |  |  |  |  |
| Normal | 1(Ref) |  | 1(Ref) |  | 1(Ref) |  |
| Overweight | 0.89(0.83 - 0.94) | <0.001 | 0.86(0.78 - 0.93) | <0.001 | 1(0.88 - 1.15) | 0.963 |
| Obesity | 1(0.94 - 1.07) | 0.98 | 0.87(0.78 - 0.96) | 0.007 | 1.48(1.28 - 1.71) | <0.001 |
| Body size change |  |  |  |  |  |  |
| Thinner→normal weight | 0.96(0.88 - 1.06) | 0.42 | 0.9(0.78 - 1.03) | 0.114 | 0.83(0.66 - 1.04) | 0.113 |
| Thinner→overweight | 0.85(0.78 - 0.93) | 0.001 | 0.8(0.7 - 0.92) | 0.001 | 1(0.82 - 1.23) | 0.987 |
| Thinner→obesity | 1.16(1.04 - 1.29) | 0.007 | 1(0.85 - 1.17) | 0.982 | 1.7(1.37 - 2.11) | <0.001 |
| Average→normal weight | 1(Ref) |  | 1(Ref) |  | 1(Ref) |  |
| Average→overweight | 0.87(0.81 - 0.95) | 0.001 | 0.82(0.73 - 0.92) | 0.001 | 0.93(0.78 - 1.12) | 0.472 |
| Average→obesity | 0.85(0.78 - 0.94) | 0.002 | 0.73(0.63 - 0.85) | <0.001 | 1.2(0.98 - 1.48) | 0.075 |
| Plumper→normal weight | 1.05(0.89 - 1.23) | 0.59 | 0.97(0.76 - 1.23) | 0.774 | 1.21(0.84 - 1.75) | 0.307 |
| Plumper→overweight | 0.94(0.83 - 1.06) | 0.303 | 0.87(0.72 - 1.04) | 0.115 | 0.87(0.65 - 1.16) | 0.351 |
| Plumper→obesity | 1.07(0.96 - 1.21) | 0.235 | 0.84(0.7 - 1.01) | 0.059 | 1.46(1.15 - 1.86) | 0.002 |

Table S6. Association of childhood body size, adulthood BMI, body size change with Dementia with covariate imputation.

|  | Dementia |  | AD |  | VD |  |
| --- | --- | --- | --- | --- | --- | --- |
|  | HR (95%CI) | P | HR (95%CI) | P | HR (95%CI) | P |
| Childhood body size |  |  |  |  |  |  |
| Average | 1(Ref) |  | 1(Ref) |  | 1(Ref) |  |
| Thinner | 1.1(1.05 - 1.15) | <0.001 | 1.09(1.02 - 1.17) | 0.012 | 1.17(1.06 - 1.29) | 0.001 |
| Plumper | 1.16(1.09 - 1.23) | <0.001 | 1.05(0.95 - 1.15) | 0.349 | 1.3(1.14 - 1.48) | <0.001 |
| Adulthood BMI |  |  |  |  |  |  |
| Normal | 1(Ref) |  | 1(Ref) |  | 1(Ref) |  |
| Overweight | 0.9(0.86 - 0.95) | <0.001 | 0.88(0.82 - 0.95) | 0.001 | 1.03(0.92 - 1.15) | 0.656 |
| Obesity | 1(0.95 - 1.06) | 0.9 | 0.87(0.8 - 0.94) | 0.001 | 1.5(1.34 - 1.69) | <0.001 |
| Body size change |  |  |  |  |  |  |
| Thinner→normal weight | 0.99(0.92 - 1.07) | 0.832 | 0.99(0.88 - 1.11) | 0.857 | 0.96(0.79 - 1.16) | 0.676 |
| Thinner→overweight | 0.93(0.86 - 1) | 0.044 | 0.9(0.81 - 1.01) | 0.067 | 1.14(0.96 - 1.35) | 0.132 |
| Thinner→obesity | 1.17(1.07 - 1.27) | 0.001 | 1.04(0.91 - 1.19) | 0.552 | 1.84(1.54 - 2.21) | <0.001 |
| Average→normal weight | 1(Ref) |  | 1(Ref) |  | 1(Ref) |  |
| Average→overweight | 0.88(0.82 - 0.94) | <0.001 | 0.87(0.79 - 0.96) | 0.005 | 0.97(0.83 - 1.14) | 0.727 |
| Average→obesity | 0.87(0.81 - 0.95) | 0.001 | 0.77(0.68 - 0.87) | <0.001 | 1.27(1.07 - 1.5) | 0.007 |
| Plumper→normal weight | 1.11(0.97 - 1.27) | 0.137 | 1.09(0.9 - 1.33) | 0.366 | 1.36(1.01 - 1.84) | 0.044 |
| Plumper→overweight | 0.99(0.89 - 1.09) | 0.795 | 0.91(0.78 - 1.06) | 0.214 | 1.05(0.83 - 1.32) | 0.688 |
| Plumper→obesity | 1.11(1.01 - 1.22) | 0.035 | 0.87(0.74 - 1.01) | 0.068 | 1.68(1.38 - 2.05) | <0.001 |
